# Cluster Analysis of Patients With Acute Ischemic Stroke: Identifying Characteristics of Long Hospital Stays in a Comprehensive Hospital

**DOI:** 10.1002/brb3.70940

**Published:** 2025-09-25

**Authors:** HeJiao Mao, Guangsong Han, Yuhui Sha, Juanjuan Wu, Mingyu Tang, Ziang Pan, Lixin Zhou, Yicheng Zhu, Jun Ni

**Affiliations:** ^1^ State Key Laboratory of Complex Severe and Rare Diseases, Department of Neurology Peking Union Medical College Hospital, Chinese Academy of Medical Sciences and Peking Union Medical College Beijing China

**Keywords:** acute ischemic stroke, age differences, cluster analysis, gender differences, in‐hospital stroke, hospital resource utilization, length of stay, NIHSS, TOAST classification

## Abstract

**Background:**

Acute ischemic stroke (AIS) is a leading cause of mortality and disability worldwide, imposing a significant burden on patients and healthcare systems. Length of stay (LOS) is a critical metric for assessing hospital resource utilization and patient prognosis. Identifying characteristics of AIS patients with prolonged LOS is essential for optimizing resource allocation and improving patient management. This study aimed to use cluster analysis to profile AIS patients with long LOS in a comprehensive hospital and explore differences in characteristics across gender and age subgroups.

**Methods:**

This single‐center, retrospective cohort study included 664 patients admitted with AIS to Peking Union Medical College Hospital from June 2012 to September 2021. Data collected included demographics, admission NIHSS scores, stroke risk factors, etiologies, and diagnostic workups. Patients were clustered using the K‐prototype method, a machine learning technique for subclassifying complex data, to differentiate between patients with long and short LOS. Statistical tests were used to identify significant differences between the clusters.

**Results:**

Cluster analysis revealed that patients with longer LOS had a higher proportion of females (42.9% vs. 24.7%, *p* < 0.001) and were generally younger (52.3 vs. 65.4 years, *p* < 0.001). This group exhibited lower proportions of TOAST type 1 strokes (17.7% vs. 70.4%, *p* < 0.001), higher levels of hsCRP and D‐dimer, and no significant difference in acute phase NIHSS scores. Notably, in‐hospital strokes and admissions to non‐neurological departments were more frequent in the long LOS group. Subgroup analysis by gender and age revealed that younger males and females shared similar characteristics with the overall long LOS group, including a higher incidence of non‐neurological department admissions and higher D‐dimer levels.

**Conclusions:**

This study highlights the heterogeneity of AIS and the importance of etiological identification, particularly in younger and female patients. Our findings suggest that traditional factors like NIHSS scores may not fully capture the complexity of factors influencing LOS in these groups. Improved cross‐departmental collaboration is crucial for better management of AIS patients.

## Graphical abstract

Cluster analysis of 664 AIS patients revealed that prolonged hospitalization is associated with female sex, younger age, higher inflammatory markers, and complex etiologies, underscoring the importance of early identification and interdisciplinary management.

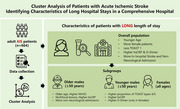

**Abbreviations**
AIS: acute ischemic stroke
CTA: computed tomography angiography
DM: diabetes mellitus
DSA: digital subtraction angiography
DWI: diffusion‐weighted imaging
EVT: endovascular therapy
IVT: intravenous thrombolysis
LOS: length of stay
MRA: magnetic resonance angiography
mRS: modified Rankin scale
NIHSS: National Institutes of Health Stroke Scale
TIA: transient ischemic attacks

## 1 Introduction

Acute ischemic stroke (AIS) is a major cause of death and disability globally, imposing significant health and economic burdens on patients and healthcare systems (Feigin et al. 2014; S. Wu et al. 2019). The heterogeneous pathophysiology of AIS leads to significant variability in clinical presentation, treatment decisions, and outcomes. An increasing number of randomized controlled trials (RCTs) have focused on acute‐phase treatment and secondary prevention for AIS, providing evidence‐based strategies to improve prognosis and reduce recurrence rates. Nonetheless, a significant proportion of patients still experience poor outcomes, influenced by factors such as age, TOAST classification, initial National Institutes of Health Stroke Scale (NIHSS) score, and time to hospital arrival. Additionally, length of stay (LOS) is a critical factor impacting both patient prognosis and healthcare costs, serving as a key indicator of hospital resource utilization and overall patient management outcomes. Precisely identifying the characteristics of AIS patients with prolonged LOS is essential for improving patient management efficiency and targeted prevention, which is vital for optimizing resource allocation within hospitals.

Previous studies have shown that factors associated with prolonged LOS in AIS patients include age, hypertension, diabetes, atrial fibrillation, previous stroke history, admission NIHSS score, and hemorrhagic transformation of stroke (Roy et al. 2024; Szczuchniak et al. 2017; Yang et al. 2023). Although some of these studies have large sample sizes, most are single‐center, retrospective cohort studies with populations that tend to be older (mean age over 67 years). Moreover, these studies primarily assess the independent effect of each factor on LOS. While important, this approach may not fully capture the complexity and heterogeneity of AIS, given its diverse etiologies and pathogenesis. There is a gap in research that comprehensively evaluates the multifactorial influences on LOS and conducts a thorough analysis of patient characteristics across different age and gender subgroups. Cluster analysis, a machine learning technique for subclassifying complex data, is a promising approach for adequately categorizing a heterogeneous disease. As an exploratory analytical method, cluster analysis is widely used in clinical big data to distinguish subtypes of complex diseases (Guo et al. 2017; Ahlqvist et al. 2018; Qi et al. 2022). This study aims to use clustering methods to characterize patients with prolonged LOS due to AIS in a comprehensive hospital and explore the differences in characteristics among gender and age subgroups.

## 2 Methods

### 2.1 Study Population

This single‐center, retrospective cohort study included adult patients with AIS admitted to Peking Union Medical College Hospital (PUMCH) between June 2012 and September 2021. PUMCH serves as a national referral center known for its specialized care of complex and rare conditions. The diagnosis of AIS was confirmed by clinicians based on the World Health Organization's clinical criteria (ICD‐11 For Mortality And Morbidity Statistics n.d.). We extracted data on patient demographics, stroke characteristics, medical history, laboratory findings, in‐hospital care measures, and discharge outcomes. This study was approved by the ethical committee of PUMCH (approval number I‐24PJ0322).

### 2.2 Data Collection

Neurological assessments at the time of admission and discharge were conducted using the NIHSS and the modified Rankin scale (mRS). Stroke subtypes were classified according to the TOAST criteria, determined by at least two experienced neurologists who reviewed comprehensive patient records. TOAST classifications at discharge included (1) large‐artery atherosclerosis, (2) cardioembolism, (3) small‐vessel occlusion, (4) stroke of other determined etiologies, and (5) stroke of undetermined etiology. All patients underwent brain imaging. Acute infarct lesions were evaluated using head computed tomography or diffusion‐weighted imaging (DWI). The decision to perform cerebrovascular imaging, including digital subtraction angiography (DSA), computed tomography angiography (CTA), or magnetic resonance angiography (MRA), was made by the attending physician according to medical needs. Imaging features, including lesion location and pattern, were assessed by at least two experienced neurologists who were blinded to the clinical details. Lesion patterns were categorized as single or involving multiple territories.

Medical history was reviewed to evaluate vascular risk factors, including hypertension, diabetes mellitus (DM), hyperlipidemia, atrial fibrillation, coronary heart disease, and smoking history. Given the focus on the acute phase of stroke, we specifically selected high‐sensitivity C‐reactive protein (hsCRP) and D‐Dimer levels to reflect the inflammatory status and fibrinolytic activity of patients. Both tests are routinely performed for most acute ischemic stroke admissions. D‐dimer is included in standard coagulation panels to guide antithrombotic therapy, evaluate perioperative/interventional risk, and aid etiological screening, while CRP is widely used to detect infection, assess systemic inflammation, and support therapeutic decisions.

Data on standard acute stroke treatment measures, including antiplatelet therapy, anticoagulant therapy, and statin therapy of any intensity, were collected. Reperfusion therapies included intravenous thrombolysis and endovascular therapy. Intravenous thrombolysis (IVT) was defined as the administration of IV tissue plasminogen activator within 4.5 h from the last known well. Endovascular therapy (EVT) was defined as thrombectomy or stent implantation performed within 24 h from the last known well.

### 2.3 Statistical Analysis

The data were normalized and hierarchically clustered using the K‐prototype clustering method, clustering method, suitable for mixed (continuous and categorical) data. Missing data for all variables were under 5% and were handled using multiple imputation with the random forest method. Features to be included in the clustering process were meticulously selected by our senior stroke experts, considering the nature of our comprehensive hospital and based on previous analyses of our stroke patient population (Wang et al. 2024; J. Wu et al. 2023; Liu et al. 2022; Tang et al. 2020, 2022; Niu et al. 2014). Various combinations of features were tested to ensure optimal clustering results. Ultimately, the most representative features, those with the greatest impact on clustering outcomes, were chosen for the final analysis. The variables included were age, gender, department of admission, whether the stroke was in‐hospital or out‐of‐hospital, TOAST classification, NIHSS score at admission, lesion type, hsCRP, and D‐Dimer levels. Initially, clustering was conducted for the entire patient cohort, followed by subgroup clustering based on age (with a cutoff at 50 years) and gender. As there is no universally accepted cut‐off for defining short versus long hospital stay in acute ischemic stroke, we applied an unsupervised clustering approach to allow the data itself to determine grouping, thereby reducing bias and better reflecting real‐world heterogeneity; accordingly, we predefined two clusters to distinguish between patients with long and short LOS, consistent with the elbow method in most subgroups except the young female group. The results are presented as mean (SD) for continuous variables and as absolute numbers (%) for categorical data. The characteristics between the long LOS and short LOS groups were compared after clustering. The means of continuous variables were compared using independent *t*‐tests, and chi‐square tests were employed to assess differences among clusters for categorical variables. The level of significance was set at two‐tailed *α* = 0.05. All statistical analyses and visualizations were performed using the R software (version 4.3.2; R Foundation for Statistical Computing, Vienna, Austria).

## 3 Results

### 3.1 Description of the Study Population

**FIGURE 1:**
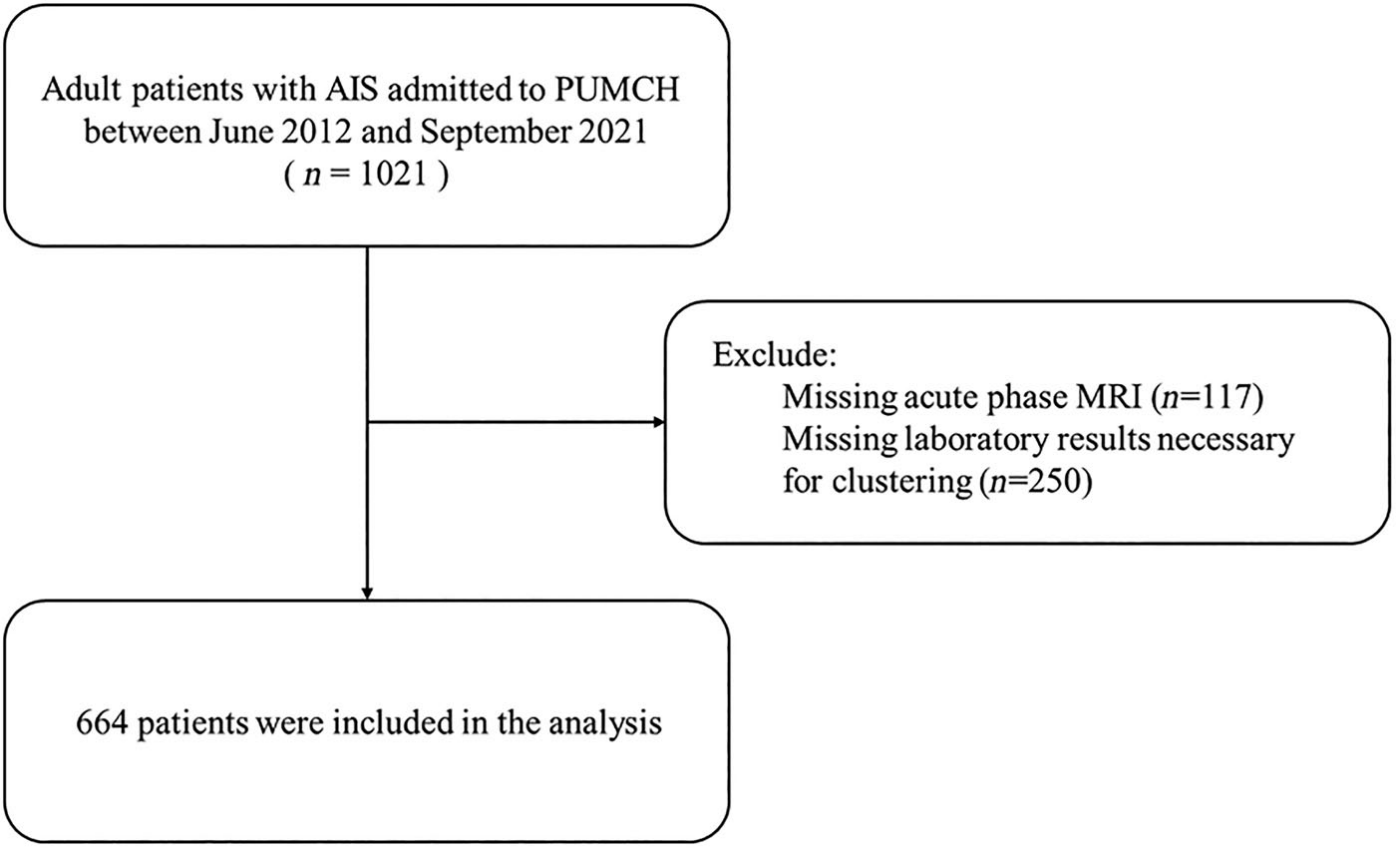
Study population.

From June 2012 to September 2021, a total of 1021 adult patients with acute stroke were admitted to our hospital. Of these, 117 patients were excluded due to missing acute‐phase MRI data, and 250 patients were excluded due to missing laboratory results essential for clustering, leaving 664 patients for the final analysis (Figure 1), ensuring that the missing data for all variables were ≤5%. As Table 1 shows, A total of 664 patients were included, with a mean age of 60.9 years; 508 were older than 50 years, and 69% were male. The neurology department admitted 337 patients (50.8%). The majority of strokes (550, 82.8%) occurred outside the hospital. TOAST type 1 was the most frequent stroke subtype (346, 52.1%), followed by types 2 (115, 17.3%) and 4 (100, 15.1%). The average NIHSS score on admission was 5.7, and the mean LOS was 18.8 days.

**TABLE 1:** Characteristics of the overall population.

	All (*n* = 664)
Clustering characteristics	
Male (%)	458 (69.0)
Age (mean [SD])	60.9 (14.7)
Admission department (%)	
Neurology	337 (50.8)
Other non‐surgical department	254 (38.3)
Surgical department	73 (11.0)
Onset = out‐of‐hospital (%)	550 (82.8)
TOAST classification (%)	
1	346 (52.1)
2	115 (17.3)
3	47 (7.1)
4	100 (15.1)
5	56 (8.4)
NIHSS at admission (mean [SD])	5.7 (5.1)
lesion type = single (%)	340 (51.2)
hsCRP (mean [SD])	16.33 (33.40)
D‐Dimer (mean [SD])	1.76 (4.40)
Outcomes	
LOS (mean [SD])	18.8 (12.7)
NIHSS at discharge (mean [SD])	3.0 (3.8)
mRS at discharge (mean [SD])	2.1 (1.5)
Medical history	
Smoking (%)	321 (48.3)
Drinking (%)	223 (33.6)
Past stroke (%)	
No	499 (75.2)
Yes	158 (23.8)
TIA	7 (1.1)
Hypertension (%)	427 (64.3)
Coronary heart disease (%)	83 (12.5)
DM (%)	227 (34.2)
Hyperlipedemia (%)	141 (21.3)
Family history (%)	159 (23.9)
Atrial fibrillation (%)	
No	605 (91.1)
Persistent	24 (3.6)
Paroxysmal	35 (5.3)
Treatment	
Antiplatelet (%)	
No	146 (22.0)
Single	405 (61.1)
Dual	112 (16.9)
Anticoagulant (%)	70 (10.5)
Statin (%)	
No	170 (25.7)
Normal	334 (50.5)
Intensive	157 (23.8)
IVT (%)	38 (5.7)
EVT (%)	
No	645 (97.1)
Thrombectomy	14 (2.1)
Stent implantation	5 (0.8)

Abbreviations: DM = diabetes mellitus; EVT = endovascular therapy; IVT = intravenous thrombolysis; LOS = length of stay; mRS = modified Rankin scale; NIHSS = National Institutes of Health Stroke Scale; TIA = transient Ischemic attacks.

### 3.2 Cluster Analysis of All Patients

Using an unsupervised clustering approach, we divided patients into two distinct groups based on LOS. One group, designated as the short LOS group, had an average LOS slightly below the overall mean (17.2 days). The other, identified as the long LOS group, had an average LOS of 21.7 days, significantly longer than the short LOS group. In the overall clustering, the long LOS group had a hospital stay approximately 4 days longer than the short LOS group (Table 2). The long LOS group had a higher proportion of females (42.9% vs. 24.7%, *p* < 0.001) and a younger average age (52.3 vs. 65.4 years, *p* < 0.001). This group showed a lower proportion of TOAST type 1 strokes (17.7% vs. 70.4%, *p* < 0.001) and higher hsCRP and D‐dimer levels (*p* < 0.001). No significant difference in acute phase NIHSS scores was found. In this comprehensive hospital, the long LOS group had a significantly higher proportion of in‐hospital strokes (24.2% vs. 8.8%, *p* < 0.001) and a majority (87%) admitted to the non‐neurology departments, while 70.9% of the short LOS group were admitted to the neurology department. Variables not included in the cluster analysis showed that the long LOS group generally had fewer vascular risk factors and a higher proportion of anticoagulant treatment.

**TABLE 2:** Cluster results of All patients.

	Long LOS (*n* = 231)	Short LOS (*n* = 433)	*p* value
Clustering characteristics			
Male (%)	132 (57.1)	326 (75.3)	<0.001*
Age (mean [SD])	52.3 (16.2)	65.4 (11.6)	<0.001*
Admission department (%)			<0.001*
Neurology	30 (13.0)	307 (70.9)	
Other non‐surgical department	173 (74.9)	81 (18.7)	
Surgical department	28 (12.1)	45 (10.4)	
Onset = in‐hospital (%)	56 (24.2)	38 (8.8)	<0.001*
TOAST classification(%)			<0.001*
1	41 (17.7)	305 (70.4)	
2	49 (21.2)	66 (15.2)	
3	28 (12.1)	19 (4.4)	
4	88 (38.1)	12 (2.8)	
5	25 (10.8)	31 (7.2)	
NIHSS at admission (mean [SD])	5.9 (5.6)	5.5 (4.7)	0.353
Lesion type = single (%)	140 (60.6)	200 (46.2)	0.001*
hsCRP (mean [SD])	30.88 (49.02)	8.56 (16.08)	<0.001*
D‐Dimer (mean [SD])	2.87 (6.29)	1.17 (2.75)	<0.001*
Outcomes			
LOS (mean [SD])	21.7 (13.6)	17.2 (11.9)	<0.001*
NIHSS at discharge (mean [SD])	3.2 (4.66)	2.8 (3.3)	0.281
mRS at discharge (mean [SD])	2.0 (1.65)	2.1 (1.5)	0.501
Medical history			
Smoking (%)	101 (43.7)	220 (50.8)	0.097
Drinking (%)	77 (33.3)	146 (33.7)	0.989
Past stroke (%)			0.258
No	178 (77.1)	321 (74.1)	
Yes	49 (21.2)	109 (25.2)	
TIA	4 (1.7)	3 (0.7)	
Hypertension (%)	123 (53.2)	304 (70.2)	<0.001*
Coronary heart disease (%)	24 (10.4)	59 (13.6)	0.281
DM (%)	61 (26.4)	166 (38.3)	0.003*
Hyperlipedemia (%)	37 (16.1)	104 (24.0)	0.023*
Family history (%)	44 (19.0)	115 (26.6)	0.039*
Atrial fibrillation (%)			0.396
No	206 (89.2)	399 (92.1)	
Persistent	11 (4.8)	13 (3.0)	
Paroxysmal	14 (6.1)	21 (4.8)	
Treatment			
Antiplatelet (%)			<0.001*
No	85 (36.8)	61 (14.1)	
Single	117 (50.6)	288 (66.7)	
Dual	29 (12.6)	83 (19.2)	
Anticoagulant (%)	38 (16.5)	32 (7.4)	<0.001*
Statin (%)			<0.001*
No	97 (42.2)	73 (16.9)	
Normal	101 (43.9)	233 (54.1)	
Intensive	32 (13.9)	125 (29.0)	
IVT (%)	10 (4.3)	28 (6.5)	0.34
EVT (%)			0.789
No	223 (96.5)	422 (97.5)	
Thrombectomy	6 (2.6)	8 (1.8)	
Stent implantation	2 (0.9)	3 (0.7)	

*Note*: *p* < 0.05 is indicated by an asterisk.

Abbreviations: DM = diabetes mellitus; EVT = endovascular therapy; IVT = intravenous thrombolysis; LOS = length of stay; mRS = modified Rankin scale; NIHSS = National Institutes of Health Stroke Scale; TIA = transient Ischemic attacks.

### 3.3 Age and Gender Stratified Clustering

Given the heterogeneity in the population, patients were stratified by gender and age (<50 and ≥50 years) for subgroup clustering (Table 3). Among males over 50 years old, the long LOS group had older patients (70.0 vs. 64.7 years, *p* < 0.001), higher initial NIHSS scores (8.3 vs. 4.0, *p* < 0.001), a higher proportion of multifocal lesions (84% vs. 33%, *p* < 0.001), and elevated hsCRP and D‐dimer levels. These patients were also more likely to be admitted to non‐neurological medical departments (83% vs. 23%, *p* < 0.001). Among males under 50, the long LOS group showed similar characteristics in terms of admission department, lesion type, and hsCRP levels but were younger than the short LOS group (37.0 vs. 43.6 years, *p* < 0.001) and had a higher proportion of TOAST types 4 or 5 (76.4% vs. 16.9%, *p* < 0.001). Female patients across all age groups had significantly longer LOS compared to males of the same age, with characteristics in the long LOS group similar to those of younger males and higher D‐dimer levels compared to the short LOS group. Due to the small number of young female patient, further age stratification was not performed.

**TABLE 3:** Cluster results of subgroups.

	Old male			Young male			Female		
	Long LOS (*n* = 117)	Short LOS (*n* = 242)	*p* value	Long LOS (*n* = 34)	Short LOS (*n* = 65)	*p* value	Long LOS (*n* = 89)	Short LOS (*n* = 117)	*p* value
Clustering characteristics									
Age (mean [SD])	70.0 (9.0)	64.7 (8.9)	<0.001*	37.1 (7.9)	43.6 (6.2)	<0.001*	50.3 (17.6)	68.4 (11.5)	<0.001*
Admission department (%)			<0.001*			<0.001*			<0.001*
Neurology	21 (17.9)	185 (76.4)		4 (11.8)	51 (78.5)		4 (4.5)	72 (61.5)	
Other non‐surgical department	76 (65.0)	36 (14.9)		27 (79.4)	11 (16.9)		70 (78.7)	34 (29.1)	
Surgical department	20 (17.1)	21 (8.7)		3 (8.8)	3 (4.6)		15 (16.9)	11 (9.4)	
Onset = in‐hospital (%)	33 (28.2)	12 (5.0)	<0.001*	7 (20.6)	3 (4.6)	0.031*	26 (29.2)	13 (11.1)	0.002*
TOAST classification (%)			<0.001*			<0.001*			<0.001*
1	69 (59.0)	148 (61.2)		6 (17.6)	36 (55.4)		10 (11.2)	77 (65.8)	
2	3 (2.6)	68 (28.1)		1 (2.9)	16 (24.6)		2 (2.2)	25 (21.4)	
3	13 (11.1)	8 (3.3)		1 (2.9)	2 (3.1)		10 (11.2)	13 (11.1)	
4	14 (12.0)	5 (2.1)		20 (58.8)	5 (7.7)		54 (60.7)	2 (1.7)	
5	18 (15.4)	13 (5.4)		6 (17.6)	6 (9.2)		13 (14.6)	0 (0.0)	
NIHSS at admission (mean [SD])	8.3 (6.8)	4.1 (3.1)	<0.001*	6.3 (5.5)	5.6 (4.5)	0.474	5.5 (5.3)	6.2 (5.1)	0.325
Lesion type = single (%)	19 (16.2)	163 (67.4)	<0.001*	9 (26.5)	46 (70.8)	<0.001*	20 (22.5)	83 (70.9)	<0.001*
hsCRP (mean [SD])	33.02 (42.34)	5.14 (9.50)	<0.001*	24.41 (42.95)	5.23 (7.76)	0.001*	38.09 (57.11)	10.04 (18.16)	<0.001*
D‐Dimer (mean [SD])	3.42 (6.01)	0.56 (0.72)	<0.001*	1.64 (3.66)	0.59 (1.40)	0.052	3.88 (7.87)	1.64 (3.21)	0.011*
Outcomes									
LOS (mean [SD])	19.8 (12.5)	15.5 (8.4)	<0.001*	24.5 (12.5)	17.5 (10.8)	0.004*	25.6 (15.4)	18.4 (16.0)	<0.001*
NIHSS at discharge (mean [SD])	4.3 (4.7)	2.1 (2.4)	<0.001*	2.9 (3.1)	2.8 (2.6)	0.781	2.9 (4.6)	3.5 (5.0)	0.365
mRS at discharge (mean [SD])	2.6 (1.7)	1.9 (1.4)	<0.001*	2.1 (1.6)	2.1 (1.4)	0.842	1.9 (1.8)	2.0 (1.5)	0.556
Medical history									
Smoking (%)	69 (59.0)	162 (66.9)	0.174	20 (58.8)	53 (81.5)	0.028*	4 (4.5)	13 (11.1)	0.146
Drinking (%)	51 (43.6)	106 (43.8)	1	16 (47.1)	39 (60.0)	0.309	6 (6.7)	5 (4.3)	0.64
Past stroke (%)			0.289			0.812			0.38
No	79 (67.5)	177 (73.1)		28 (82.4)	52 (80.0)		73 (82.0)	90 (76.9)	
Yes	38 (32.5)	63 (26.0)		5 (14.7)	12 (18.5)		14 (15.7)	26 (22.2)	
TIA	0 (0.0)	2 (0.8)		1 (2.9)	1 (1.5)		2 (2.2)	1 (0.9)	
Hypertension (%)	75 (64.1)	173 (71.5)	0.195	9 (26.5)	38 (58.5)	0.005*	39 (43.8)	93 (79.5)	<0.001*
Coronary heart disease (%)	25 (21.4)	28 (11.6)	0.022*	2 (5.9)	1 (1.5)	0.562	9 (10.1)	18 (15.4)	0.367
DM (%)	41 (35.0)	92 (38.0)	0.667	8 (23.5)	18 (27.7)	0.836	17 (19.1)	51 (43.6)	<0.001*
Hyperlipedemia (%)	24 (20.7)	61 (25.2)	0.419	3 (8.8)	15 (23.1)	0.141	7 (7.9)	31 (26.5)	0.001*
Family history (%)	25 (21.4)	71 (29.3)	0.141	8 (23.5)	20 (30.8)	0.6	7 (7.9)	28 (23.9)	0.004*
Atrial fibrillation (%)			0.258			1			0.517
No	103 (88.0)	225 (93.0)		0 (0.0)	0 (0.0)		80 (89.9)	99 (84.6)	
Persistent	7 (6.0)	10 (4.1)		0 (0.0)	0 (0.0)		2 (2.2)	5 (4.3)	
Paroxysmal	7 (6.0)	7 (2.9)		0 (0.0)	1 (1.5)		7 (7.9)	13 (11.1)	
Treatment									
Antiplatelet (%)			0.076			0.049*			<0.001*
No	27 (23.1)	34 (14.1)		10 (29.4)	7 (10.8)		49 (55.1)	19 (16.2)	
Single	65 (55.6)	159 (66.0)		19 (55.9)	41 (63.1)		36 (40.4)	85 (72.6)	
Dual	25 (21.4)	48 (19.9)		5 (14.7)	17 (26.2)		4 (4.5)	13 (11.1)	
Anticoagulant (%)	17 (14.5)	12 (5.0)	0.004*	3 (8.8)	4 (6.2)	0.937	26 (29.2)	8 (6.8)	<0.001*
Statin (%)			0.666			0.056			<0.001*
No	24 (20.5)	42 (17.5)		14 (41.2)	12 (18.8)		57 (64.0)	21 (17.9)	
Normal	62 (53.0)	125 (52.1)		14 (41.2)	35 (54.7)		26 (29.2)	72 (61.5)	
Intensive	31 (26.5)	73 (30.4)		6 (17.6)	17 (26.6)		6 (6.7)	24 (20.5)	
IVT (%)	11 (9.4)	13 (5.4)	0.227	1 (2.9)	7 (10.8)	0.333	0 (0.0)	6 (5.1)	0.08
EVT (%)			<0.001*			0.085			0.165
No	108 (92.3)	241 (99.6)		0 (0.0)	0 (0.0)		88 (98.9)	114 (97.4)	
Thrombectomy	6 (5.1)	0 (0.0)		4 (11.8)	1 (1.5)		0 (0.0)	3 (2.6)	
Stent implantation	3 (2.6)	1 (0.4)		0 (0.0)	0 (0.0)		1 (1.1)	0 (0.0)	

*Note*: *p* < 0.05 is indicated by an asterisk.

Abbreviations: DM = diabetes mellitus; EVT = endovascular therapy; IVT = intravenous thrombolysis; LOS = length of stay; mRS = modified Rankin scale; NIHSS = National Institutes of Health Stroke Scale; TIA = transient Ischemic attacks.

## 4 Discussion

This study is the first to use a machine learning–based clustering analysis to comprehensively evaluate the factors influencing LOS in patients with AIS at a single comprehensive hospital, integrating both clinical and imaging data. We identified distinct clusters of patients with AIS with prolonged LOS, characterized by a higher prevalence of females, younger age, higher hsCRP and D‐dimer levels, and a lower proportion of TOAST type 1 strokes. Notably, in‐hospital strokes and admissions to non‐neurological departments were more common in the long LOS group. In contrast, NIHSS scores did not consistently influence LOS across all AIS patients. These findings highlighted the significant impact of the etiological complexity of stroke on LOS, surpassing the effect of baseline NIHSS scores.

Prior studies on the AIS population at the same center have underscored the impact of complex stroke etiologies on patient prognosis and treatment strategies (Wang et al. 2024; J. Wu et al. 2023; Liu et al. 2022; Tang et al. 2020, 2022; Niu et al. 2014). Our study further demonstrates their influence on LOS. For in‐patients in a comprehensive hospital, the time required for thorough etiological investigations is a key determinant of total LOS. A high NIHSS score does not necessarily prolong the hospital stay. Patients may opt to transfer to rehabilitation facilities after the acute phase for continued recovery and secondary prevention treatments. Previous cluster analyses in AIS populations have focused on single examinations or influencing factors, such as electrocardiogram, computed tomography, or biological samples (Baumgartner et al. 2005; Tsunetoshi et al. 2023; Zhang et al. 2018). A recent study applied clustering methods to group patients with AIS and identified factors associated with poor functional recovery. These included age, systolic blood pressure, pulse, D‐dimer levels, low‐density lipoprotein levels, hemoglobin, creatinine levels, NIHSS scores, and Barthel Index scores at admission (Hajiesmaeili et al. 2024). LOS is frequently used as an outcome measure in studies evaluating intervention effectiveness. It is closely linked to prognosis and hospitalization costs of patients with AIS and also reflects the efficiency of healthcare resource utilization (Y. Wu et al. 2021). Reducing LOS can greatly enhance the efficiency of medical resource utilization. The result suggests that traditional factors such as NIHSS may not fully capture the complexity of factors influencing LOS, especially in younger and female patients. The close association of prolonged LOS with younger age, female sex, elevated inflammatory markers, and atypical TOAST classifications underscores the pivotal role of complex stroke etiologies in prolonging LOS, which directly increases treatment costs and potentially contributes to poorer outcomes. Emphasis should be placed on etiological identification and management, particularly for non‐neurological department stroke cases, highlighting the need for interdisciplinary collaboration. Among these inflammatory markers, CRP is particularly noteworthy. As a systemic inflammatory protein, elevated CRP may indicate infection, systemic inflammation, or complex stroke mechanisms and complications, all of which can lead to unfavorable outcomes and extended hospitalization (Napoli et al. 2001; Muir et al. 1999; Bian et al. 2023). Given its accessibility and reproducibility in routine practice, monitoring CRP provides valuable prognostic information and has been consistently validated in previous studies of acute ischemic stroke patients.

Differences observed between the subgroups may be attributed to variations in stroke mechanisms and risk factors. Among older male patients, those with a greater age and more severe stroke (e.g., more lesions and higher NIHSS scores) tend to have longer LOS. The long LOS group also had a higher prevalence of coronary artery disease, while there was no significant difference in other chronic diseases compared to the short LOS group. This may be due to their high prevalence and relatively uniform distribution among elderly patients with stroke in China (Lu et al. 2017; Tu et al. 2022). In younger males, the long LOS group was characterized by younger age, atypical stroke types, and a systemic inflammatory response, potentially reflecting underlying conditions such as autoimmune diseases or hereditary thrombophilia, which require more comprehensive etiological screening and thus prolonged hospitalization. Female patients across all age groups exhibited longer LOS, which may be related to gender‐specific risk factors, such as hormonal influences or differences in thrombotic tendencies. Previous studies have also indicated that gaps in awareness of stroke in women, less frequent use of diagnostic tests, and other sociodemographic factors can affect stroke prognosis in females (Bushnell et al. 2018; Di Carlo et al. 2003). Unfortunately, due to the limited sample size and the analysis methods requiring handling of missing data, we could not further explore the impact of sex and related factors, such as contraceptive use, on LOS. In fact, the interaction between sex and age reveals a complex pattern in terms of stroke hospitalization duration, warranting further investigation.

A major strength of this study is its use of cluster analysis, a machine learning approach that allows for the identification of patient subgroups with distinct profiles that are more representative of real‐world scenarios. By simultaneously considering multiple variables—such as age, sex, stroke type, inflammation markers, and clinical characteristics—this method provides a more nuanced understanding of how these factors interact and collectively influence LOS. This approach moves beyond traditional single‐variable analysis, revealing complex patterns and interactions that might otherwise remain undetected. As there is currently no universally recognized cut‐off value for LOS in stroke populations, this strategy avoids the arbitrariness of pre‐specified thresholds and better captures the heterogeneity of real‐world clinical practice. This study has several limitations, primarily related to the patient population. First, the hospital receives a large number of patients with complicated conditions, and the neurology department itself has a selection bias in the patients it treats. This may lead to further complexity in the etiologies of strokes and limits the generalizability of the study's findings. For example, the mean NIHSS score in our cohort was relatively low because patients with very large territorial infarctions or malignant edema requiring urgent neurosurgical intervention are typically admitted to regional hospitals with stronger neurosurgical capacity. Our hospital more often receives patients with moderate severity, complex etiologies, or those transferred for further evaluation. Additionally, the retrospective nature of the study may introduce biases related to data availability and accuracy. Our clustering intentionally focused on admission variables to enable early stratification. Post‐stroke complications were not incorporated after clustering, which limited the explanatory dimension of the identified phenotypes. Future research should aim to validate these findings through prospective, multicenter studies with predefined outcomes in similar comprehensive hospital populations, and to explore the utility of additional biomarkers and imaging modalities in refining patient stratification and management strategies.

## 5 Conclusion

In general, this study utilized cluster analysis to identify distinct characteristics of AIS patients with prolonged LOS in a comprehensive hospital setting. The findings highlight significant gender and age differences in the factors influencing LOS, underscoring the importance of comprehensive etiological assessment and interdisciplinary collaboration in stroke management.

## Author Contributions

HeJiao Mao conceptualized the study, conducted the primary analysis, and drafted the manuscript. Guangsong Han, Yuhui Sha, Juanjuan Wu, Mingyu Tang, Ziang Pan, and Lixin Zhou contributed to data collection, organization, and the verification of patient diagnoses. Yicheng Zhu and Jun Ni provided guidance on the overall research direction, with Jun Ni reviewing and revising the manuscript. All authors read and approved the final manuscript.

## Ethics Statement

This study was approved by the ethical committee of PUMCH (approval number I‐24PJ0322).

## Conflicts of Interest

The authors declare no conflicts of interest.

## Peer Review

The peer review history for this article is available at https://publons.com/publon/10.1002/brb3.70940.

## Data Availability

The datasets generated and/or analyzed during the current study are not publicly available because the data were obtained from a retrospective review of medical records and contain sensitive patient information, but are available from the corresponding author on reasonable request.
